# 100% Conversion of CO_2_–CH_4_ with Non-Precious Co@ZnO Catalyst in Hot Water

**DOI:** 10.1007/s40820-025-01711-6

**Published:** 2025-04-14

**Authors:** Yang Yang, Xu Liu, Daoping He, Fangming Jin

**Affiliations:** 1 School of Environmental Science and Engineering, Shanghai Jiao Tong University, Shanghai, 200240 People’s Republic of China; 2 China-UK Low-Carbon College, Shanghai Jiao Tong University, Shanghai, 200240 People’s Republic of China; 3 Shanghai Key Laboratory of Hydrogen Science & Center of Hydrogen Science, State Key Laboratory of Metal Matrix Composites, Shanghai Jiao Tong University, Shanghai, 200240 People’s Republic of China; 4 School of Environmental Science and Engineering, Hainan University, Haikou, 570228 People’s Republic of China

**Keywords:** CO_2_ methanation, Cobalt catalyst, Hydrothermal, Formic acid, Co@ZnO catalyst

## Abstract

The combination of solar energy and natural hydrothermal systems will innovate the chemistry of CO_2_ hydrogenation; however, the approach remains challenging due to the lack of robust and cost-effective catalytic system. Here, Zn which can be recycled with solar energy-induced approach was chosen as the reductant and Co as catalyst to achieve robust hydrothermal CO_2_ methanation. Nanosheets of honeycomb ZnO were grown in situ on the Co surface, resulting in a new motif (Co@ZnO catalyst) that inhibits Co deactivation through ZnO-assisted CoO_x_ reduction. The stabilized Co and interaction between Co and ZnO functioned collaboratively toward the full conversion of CO_2_–CH_4_. In situ hydrothermal infrared spectroscopy confirmed the formation of formic acid as an intermediate, thereby avoiding CO formation and unwanted side reaction pathways. This study presents a straightforward one-step process for both highly efficient CO_2_ conversion and catalyst synthesis, paving the way for solar-driven CO_2_ methanation.

**Supplementary Information:**

The online version contains supplementary material available at 10.1007/s40820-025-01711-6.

## Highlights

- The combination of solar energy and underground hydrothermal environment supports the sustained and efficient CH_4_ production from CO_2_.
- Nanosheets of honeycomb ZnO were formed in-situ on the Co surface, resulting in a new motif (Co@ZnO catalyst) that inhibits Co deactivation through ZnO-assisted CoOx reduction.
- The stabilized Co and interaction between Co and ZnO inhibited unwanted side reaction pathways via CO production, ensuring formic acid formed as an intermediate, leading to 100% CH_4_ yield.

**Supplementary Information:**

The online version contains supplementary material available at 10.1007/s40820-025-01711-6.

## Graphical abstract

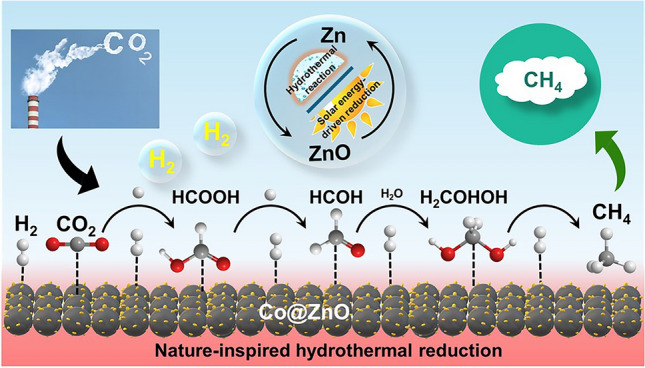

## Introduction

Access to affordable and reliable energy that is produced with minimal negative environmental impacts represents a global challenge. To this end, the conversion of carbon dioxide (CO_2_), which is the main greenhouse gas, into hydrocarbons using solar energy is critical for minimizing these environmental issues. The photocatalytic conversion of CO_2_ over semiconductors has been widely developed and reported [1, 2]; however, the efficiency of the conversion is limited by the kinetic restrictions of multiple e^−^/H^+^ transfer processes and ability of the semiconductors to activate thermodynamically stable CO_2_ [3, 4]. In contrast, the cascade approach for CO_2_ conversion by solar energy has sparked interest in exploring novel methods for fuel generation. This approach involves a two-step process in which active reductants such as H_2_ or metals are initially produced via a solar energy-driven reaction, followed by the conversion of CO_2_ utilizing the reductive power of hydrogen or active metals [5, 6]. These two-step solar-chemical approaches employ diverse and robust reductants as the driving force, leading to higher reaction rates, flexibility in the selection of non-noble metal-based catalysts, and the potential for large-scale industrial applications.

In pioneering work by A. Steinfeld, the heat obtained from concentrated solar radiation was used to drive multiple cycles of metal oxide redox pairs, including Zn/ZnO, Ce_2_O_3_/CeO_2_, FeO/Fe_3_O_4_, and SnO/SnO_2_, potentially offering an abundant selection of reductants for CO_2_ conversion [7–12]. Further insights into metal-initiated CO_2_ reduction are gained from abiogenic organic synthetic processes. In bio- and geo-chemistry, abiogenic organic compounds are formed from CO_2_ reduction in the mantle and submarine hydrothermal vents, in which serpentinization delivers a substantial amount of hydrogen from the interaction between high-temperature water and earth-abundant metals [13–16]. By mimicking this natural phenomenon, we demonstrated efficient CO_2_ reduction under hydrothermal conditions using metals Zn, Fe or Al as reductants [17–19], with Zn showing the highest CO_2_ reduction efficiency [20, 21], thereby standing as a promising reductant for deeper CO_2_ reduction processes, such as CO_2_ methanation (CO_2_ + 4H_2_ → CH_4_ + 2H_2_O, Sabatier reaction). Since methane (CH_4_) is an ideal energy carrier and hydrocarbon fuel can be conveniently distributed using well-developed gas pipeline infrastructure [22–28], the CO_2_–CH_4_ cycle could potentially close the carbon fuel emission cycle. Thus, we consider CH_4_ production ideal for energy storage and grid integration, providing a practical approach to utilizing CO₂, especially in the context of renewable energy.

In abiogenic organic synthesis, the origin of life also lies in the unique catalytic role of common earth-abundant rocks [29–32]. For instance, the acetyl-CoA pathway, which is the only known exergonic autotrophic CO_2_ fixation pathway, is actively catalyzed by the bifunctional carbon monoxide dehydrogenase/acetyl-CoA synthase (CODH/ACS). In this enzyme, transition metal (Ni, Fe) sulfide clusters occupy the active sites, and Co functions as the core element for catalyzing the reduction in formal groups to methyl species [33, 34], indicating Co-based catalysts to exhibit high catalytic activity for CO_2_ methanation particularly under hydrothermal conditions. Despite the potential of Co-based catalysts for CH_4_ production, they are easily oxidized and deactivated when exposed to wet vapor [35, 36]. Recently, we found that Co can be stabilized by bicarbonate-assisted CoO_x_ reduction in a hydrothermal environment; however, partial CO_2_/NaHCO_3_ was used as the stabilizer leading to CoCO_3_ formation, thereby impeding the full conversion of CO_2_/NaHCO_3_ to hydrocarbon. In addition, hydrocarbons beyond C_2_ were also formed through a CO intermediate, decreasing the selectivity for CH_4_ [17]. Therefore, efficient CO_2_ methanation is looking for a promising strategy to stabilize Co-based catalysts.

**Fig. 1:**
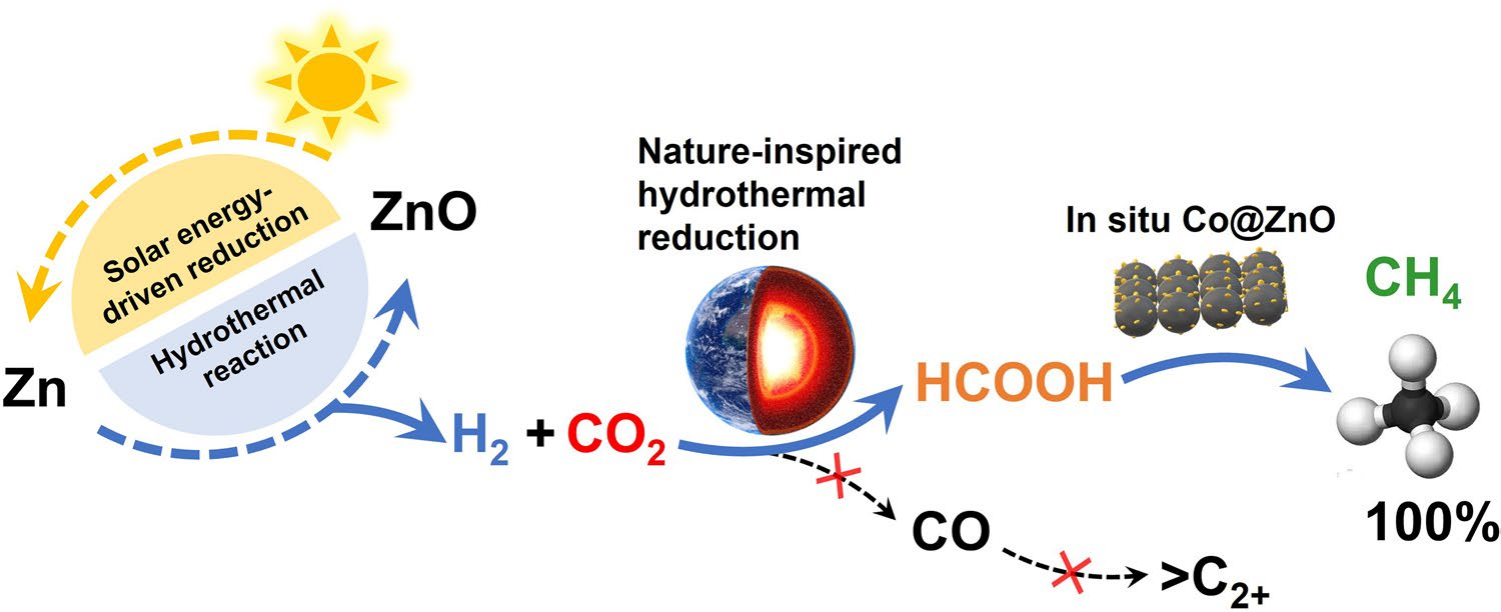
Schematic illustration of an integrated system that combines solar energy aboveground and a hydrothermal environment underground for sustained and efficient methane (CH_4_) production from carbon dioxide (CO_2_) using an Co@ZnO catalyst

Here, we report the hydrothermal-based methanation of CO_2_ using the simple metals such as Zn and Co. Notably, Co remained in a metallic state throughout the reaction due to the rigid reductive atmosphere provided by Zn and the refresh process of ZnO activating in situ formed H_2_. Local honeycomb ZnO nanosheets were formed on the Co surface, leading to the formation of Co@ZnO catalyst. The presence of zero-valent Co and Co–ZnO interaction resulted in CH_4_ yield from CO_2_ reduction of up to 100%, which was achieved by thoroughly inhibiting the formation of CO intermediate and enhanced adsorption of CO_2_ and intermediate formic acid. The development of an integrated solar-hydrothermal Sabatier reaction involving Zn and Co advances the use of Co-based catalyst in the chemical engineering field, and may provide a simple, sustainable, and highly efficient approach for CO_2_ methanation (Fig. 1).

## Experimental Section

### Materials

Zn powder (200 mesh), Co powder (200 mesh), NaHCO_3_ (AR, ≥ 98%), and NaOH (> 96%) were purchased from Sinopharm Chemical Reagent Co., Ltd. Gaseous CO_2_ (> 99.995%) and H_2_ (> 99.995%) were purchased from Shanghai Poly-Gas Technology Co., Ltd. Deionized water (18.25 MΩ cm^−1^) was used in all experiments. All reagents were commercially available and used without further purification.

### Experimental Procedures

Hydrothermal CO_2_ methanation experiments were conducted in a stainless steel (SUS-316) batch reactor with an inner volume of 42 mL. In a typical run, the desired amount of Zn powder (30–90 mmol), Co powder (20–80 mmol), and NaOH solution (0–0.4 mol L^−1^) was added into the reactor chamber, and 1.5 MPa CO_2_ was then introduced into the reactor. (The reactor was purged with CO_2_ in advance.) The reactor was sealed and placed into an induction furnace heated to the target temperature (250–325 °C) at a rate of 15 °C min^−1^. During the reaction, the induction furnace was swayed constantly at a rate of 20 times min^−1^. After 1–5 h, the reactor was removed from the induction furnace and allowed to cool to room temperature, and gaseous, liquid and solid samples were collected for analysis.

Gaseous samples were analyzed using a gas chromatograph equipped with a thermal conductivity detector (GC–TCD, HP-5890 Series II) and an HP − 1 packing column. The GC temperature program started at 60 °C for 5 min, increased to 120 °C at 5 °C min^−1^, and was then held at 120 °C for 12 min. He was applied as the carrier gas at a flow rate of 65 mL min^−1^ to detect CO_2_ and CO, and N_2_ was applied as the carrier gas at a flow rate of 65 mL min^−1^ for detecting CH_4_ and H_2_.

Liquid products were analyzed by high-performance liquid chromatography (HPLC) and gas chromatography–mass spectrometry (GC − MS). HPLC analysis was performed on an Agilent 1200 system, which was equipped with two KC-811 columns (SHODEX) for sample separation and a tunable UV-vis absorbance detector adjusted to 210 nm for sample detection. A solution of HClO_4_ (2 mmol L^−1^) was used as the mobile phase at a flow rate of 1.0 mL min^−1^. For GC − MS analysis, a Hewlett-Packard model 7890A gas chromatograph system equipped with a model 5975C mass selective detector was used. The initial temperature of the oven in the gas chromatograph was 313 K, which was maintained for 1 min, and the temperature was then increased at a rate of 7 K min^−1^ to a final temperature of 503 K, which was held for 20 min. Samples were separated with a HP-INNOWAX polar capillary column (25 m long, 0.25 mm i.d., 0.5 μm film thickness) using helium as a carrier gas.

Solid samples were washed several times with deionized water and were then dried under vacuum conditions for 24 h. The materials were investigated by X-ray diffraction (XRD) measurements using a Bruker D8 Advance X-ray diffractometer with Cu K*α* radiation (*λ* = 1.54184 Å) and a scanning rate of 2° min^−1^ from 10° to 80° (2θ). Surface morphologies of the catalysts were analyzed using a scanning electron microscope (SEM; FEI Quanta 200) at an acceleration voltage of 5 kV. X-ray photoelectron spectroscopy (XPS) measurements were performed on a K-Alpha + XPS spectrometer system (Thermo Electron Corp., ESCALAB 250Xi, 1486.6 eV) with Al K*α* radiation. High-resolution transmission electron microscopy (HRTEM) images were obtained with a JEOL JEM-2100F at 200 kV, and a copper mesh ultra-thin carbon film was used as the sample stage. Temperature-programmed desorption of CO_2_ (CO_2_-TPD) was employed to measure the basicity of samples with Autosorb-iQ-C. The possible leaching of the catalyst was measured by inductively coupled plasma-optical emission spectrometry (ICP-OES) using a PerkinElmer Avio 500 OES.

### X-Ray Absorption Fine Structure (XAFS) Spectroscopy Measurements

XAFS spectroscopy was performed at SPring-8 (Japan Synchrotron Radiation Research Institute, Hyogo, Japan). The XAS spectra were collected in transmission mode at 8.0 GeV with a constant current of 99.5 mA. The XAFS data were processed according to the standard procedures using the Athena module implemented in the IFEFFIT software packages. The EXAFS spectra were obtained by subtracting the post-edge background from the overall absorption and then normalizing with respect to the edge-jump step. Subsequently, the *χ(k)* data of were Fourier transformed into real (R) space using a Hanning windows (dk = 1.0 Å^−1^) to separate the EXAFS contributions from different coordination shells. To obtain the quantitative structural parameters around central atoms, least-squares curve parameter fitting was performed using the ARTEMIS module of IFEFFIT software packages [37, 38]

### Computation Methods

Spin-polarized electronic structure calculations were performed using the plane-wave basis set approach as implemented in the Vienna ab initio simulation package (VASP). The projector augmented wave (PAW) method was used to represent the ion–core electron interactions. The valence electrons were represented with a plane-wave basis set with an energy cutoff of 450 eV. Electronic exchange and correlation were described with the Perdew–Burke–Ernzerhof (PBE) functional. The DFT-D3 method was used to treat van der Waals interactions. Four layers of 4 × 4 supercells of the Co (0001) surface was used as pure Co catalyst and 8 layers of 3 × 3 supercells of the ZnO (0001) surface with one layer of the Co (0001) surface was used as the Co@ZnO Co catalyst. A 15 Å vacuum space was used to avoid interactions between surface slabs. A 3 × 3 × 1 Monkhorst–Pack scheme was used to generate the k-point grid for the modeled surfaces. The convergence criteria for the self-consistent electronic structure and geometry were set to 10^−5^ eV and 0.05 eV Å^−1^, respectively. Zero-point vibrational energy (ZPVE) corrections were calculated by assuming a quantum harmonic oscillator possessing the calculated vibrational frequency. Transition state calculations were performed using the climbing image nudged elastic band (CI-NEB) method with the transition state identified to have an absolute tangent force below 0.05 eV Å^−1^.

### In situ Hydrothermal Fourier Transform Infrared (FTIR) Spectroscopy

To investigate the reaction mechanism, operando hydrothermal infrared spectroscopy was used to monitor the reaction process. The operando hydrothermal infrared spectroscopy was composed of an IR spectrometer (Thermo Fisher, iS10), observation box, high-pressure/high-temperature reactor cell equipped with a diamond window (the cell can maintain a temperature as high as 250 °C and pressure as high as 30 MPa), heating system, high pressure pump, and cooling system. A schematic drawing of the equipment is shown in Fig. S13. When monitoring the hydrothermal reaction, the desired number of reactants and water were sealed in the reactor, which was then placed into the observing box. After reaching the target reaction temperature, in situ hydrothermal FTIR spectra were collected simultaneously as the reaction proceeded.

### Separation of Co and ZnO from Co@ZnO Catalyst

Co and ZnO were separated from the Co@ZnO catalyst using the following procedure. First, the Co@ZnO catalyst and 200 mL of water were added into a beaker, which was treated with an ultrasonic machine for 10 min to separate ZnO and Co. A magnet was then used to move Co into one solid phase. ZnO was dispersed in the water as a suspended solid and was transferred with water into another beaker. The above steps were repeated several times until no ZnO was washed off from the Co@ZnO catalyst. Finally, Co was collected as one solid aggregate, whereas ZnO remained in water.

### CH_4_ Yield

The CH_4_ yield was defined as the molar ratio of the amount of carbon in CH_4_ to the initial amount of CO_2_, as shown in the following equation:

$$ {\text{CH}}_{4} {\text{yield}}/\% = \frac{{n_{{{\text{CH}}_{{4}} }} {\text{ after reaction, mmol}}}}{{n_{{{\text{CO}}_{{2}} }} {\text{initial added, mmol}}}} \times 100\% $$

## Results and Discussion

### Hydrothermal Methanation of CO_2_ with Zn and Co

The hydrothermal methanation of CO_2_ using Zn and Co was carried out in a series of stainless steel autoclaves at temperatures ranging from 250 to 325 °C. Control experiments using only Zn resulted in the formation of only formic acid. In contrast, CH_4_ was clearly detected in the presence of metallic Co. Since after the reaction, Zn was oxidized to ZnO. To isolate the catalytic effect of ZnO, CO_2_ methanation was performed with ZnO as the catalyst; however, only formic acid was observed again. On the other hand, even with Co addition, the CH_4_ yield was limited to approximately 40%, which was attributed to the restricted dissolution of gaseous CO_2_. By gradually increasing the alkalinity of the reaction solution, the CH_4_ yield increased to 77% (Fig. S1a). However, further increasing the alkalinity of solution led to the decrease in CH_4_ yield, resulting in a volcano type of relationship between the alkalinity and CH_4_ yield. To study the underlying reason, we determined the distribution of carbon species (H_2_CO_3_, HCO_3_^−^, and CO_3_^2−^) in solution at 300 °C with various alkaline concentration (details of analyzing the distribution of carbon species are provided in the SI), and a strong positive correlation was observed between CH₄ yield and the initial HCO₃⁻ concentration. This finding aligns with our previous results that HCO_3_^−^ was the most reactive carbon species for reduction under hydrothermal conditions, and with Zn as the reductant, it could be converted to formic acid most conveniently [19–21], which is just the intermediate for CH_4_ in this reaction as demonstrated later. Furthermore, the isotope experiment with ^13^CO_2_ demonstrated that the origin of CH_4_ was from CO_2_ reduction, as evidenced by GC–MS analysis (Fig. S1b).

**Fig. 2:**
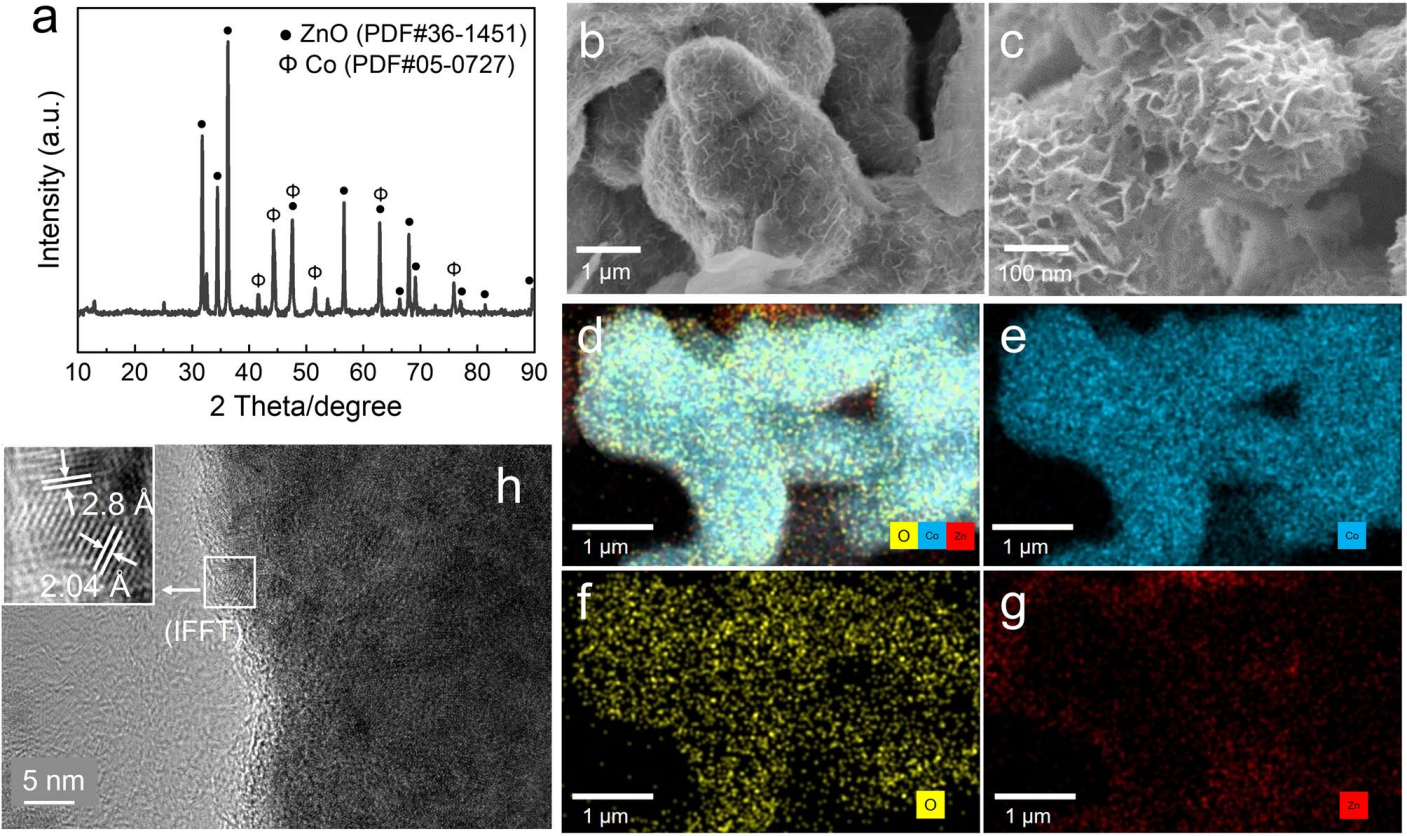
Characterizations of Co@ZnO catalyst. **a** XRD, **b**, **c** SEM images, **d**–**g** SEM–EDS analysis. **h** HRTEM images, IFFT, inverse fast Fourier transform

The solid phase of the methanation reaction was analyzed by X-ray diffraction (XRD; Fig. 2a). The peaks at 41.7°, 44.8°, 47.6°, 62.7°, and 75.9° were originated from the hexagonal closed-packed cobalt (PDF#05–0727), and the peaks at 31.7°, 34.4°, 36.3°, 47.5°, 56.6°, 62.9°, and 68.0° were assigned to ZnO (PDF#36–1451), indicating that Zn was oxidized to ZnO for hydrogen production, whereas Co remained in a native valence state. SEM images of the catalyst showed a thin nanosheet growing around nanorods structure (Fig. 2b, c). In the enlarged image, the observed nanosheets had a honeycomb-like motif with a thickness of ~ 10 nm. Energy-dispersive X-ray (EDX) elemental mapping analysis was conducted to identify the elementary distribution. As shown in Fig. 2d–g, the nanorods were comprised of Co, whereas O and Zn constituted the nanosheets surrounding the nanorods, indicating that a Co-supported ZnO honeycomb nanosheet structure was formed. To further understand the elementary structure of the solid sample, ZnO was removed from the collected solid sample through ultrasonic treatment. With most of the ZnO cleaned off from Co surface (Fig. S2a), XRD analysis confirmed that metallic Co remained (Fig. S2b). The structure of the solid remain was further investigated by HRTEM (Fig. 2h). Rod-shaped Co crystals with regular lattice fringes and a well-defined crystallite were observed. The d-spacing of Co was 2.04 Å, which agrees with the (111) interlayer spacing of FCC-Co. Furthermore, ZnO with a d-spacing of 2.8 Å, which corresponded to the (100) spacing of wurtzite-ZnO, existed at the Co edge. In addition, disordered lattices were observed in the contact zone of Co and ZnO in the IFFT patterns. Collectively, these results demonstrated that Co-supported honeycomb-like ZnO nanosheets (Co@ZnO) were successfully fabricated with a Co–ZnO interface.

**Fig. 3:**
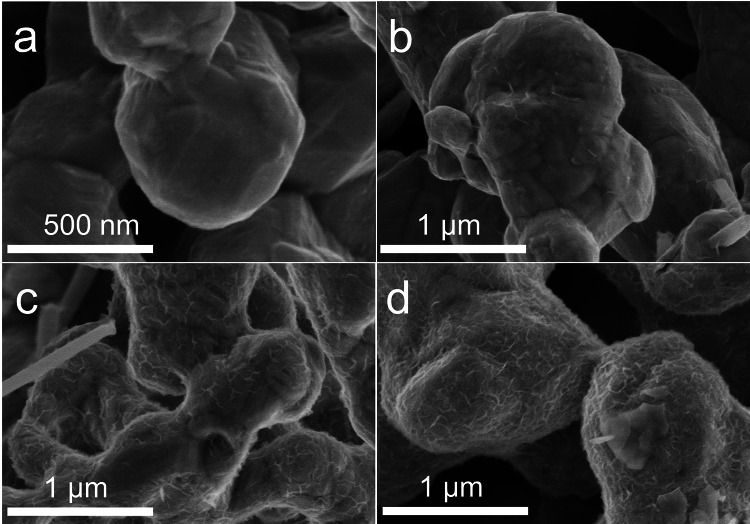
Time-dependent SEM images of Co@ZnO in the temporal experiments of CO_2_ methanation by Zn and Co under hydrothermal conditions. **a** 5 min, **b** 10 min, **c** 30 min, **d** 1 h (reaction conditions: 280 °C, 60 mmol Zn, 40 mmol Co, 1.5 MPa CO_2_, 0.1 mol/L NaOH, 280 °C was selected deliberately to slow down the growth of Co@ZnO catalyst)

The growth of ZnO on the Co surface of Co@ZnO was further examined through time-resolved experiments. SEM images of solid samples collected at various time intervals revealed the progression of ZnO formation, alongside tracking CH_4_ production. Initially, Co was rod shaped with a smooth surface (Fig. 3a), while honeycomb ZnO nanosheets began to form on the Co surface after a reaction time of 10–30 min (Fig. 3b, c) and continued to grow as the reaction proceeded for 1 − 3 h (Fig. 3d). Concurrently, CH_4_ production showed a sharp increase from 30 min to 1 h and continued accumulating over the next 2 h (Fig. S3), indicating that the formation of the Co@ZnO catalyst significantly enhances CH_4_ generation.

### Maintenance of Co in a Zero-Valence State in the Zn–Co Hydrothermal System

**Fig. 4:**
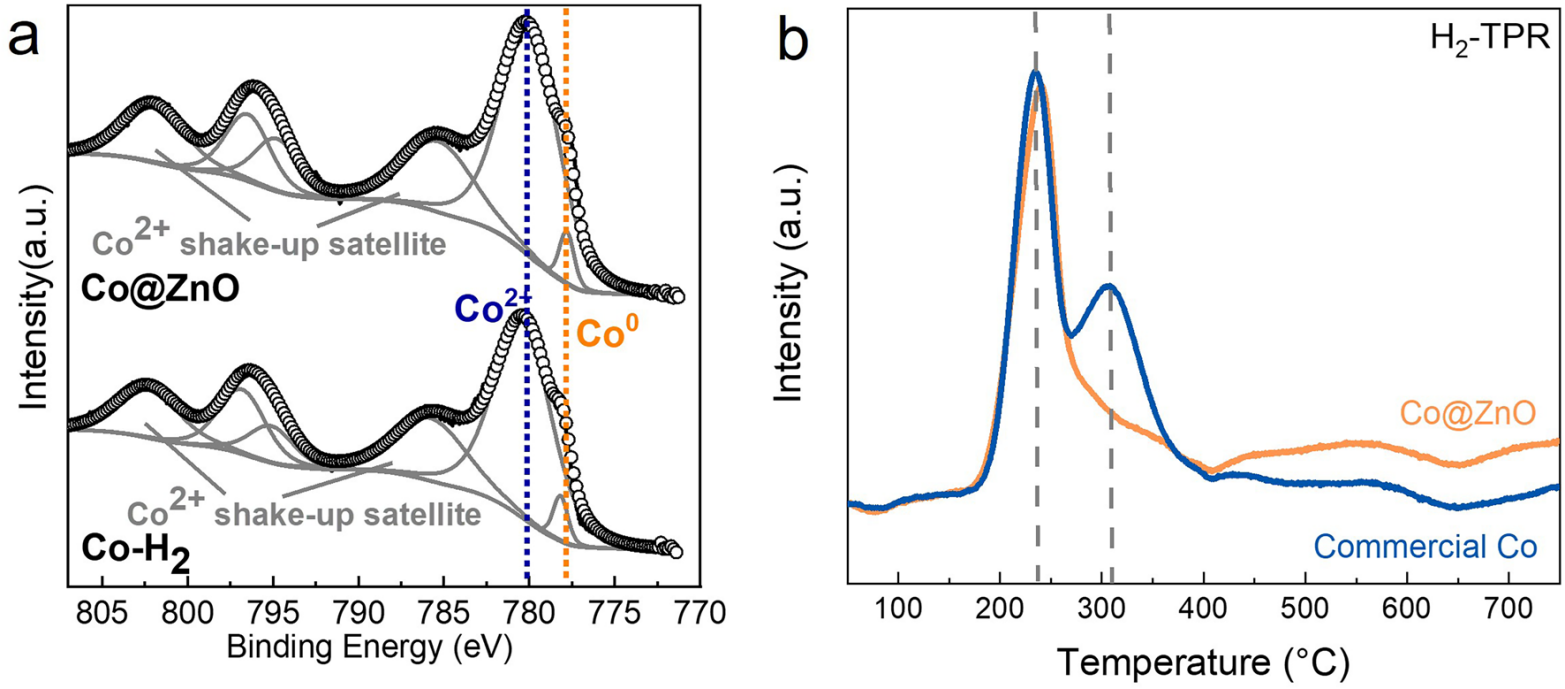
Characterizations of Co@ZnO catalyst and commercial Co. **a** XPS spectra of Co@ZnO and pre-reduced commercial Co. **b** H_2_-TPR analysis of Co@ZnO and commercial Co exposed to air

The chemical state of the Co@ZnO surface was further studied with XPS analysis (Fig. 4). For comparison, commercial Co pre-reduced (Co–H_2_) was also analyzed. As shown in Fig. 4a, CoO and Co coexisted on both the Co@ZnO catalyst and Co–H_2_. The existence of the CoO signal on the two samples was likely due to oxidation of the Co surface in air prior to the XPS analysis. We then explored how Co retains its metallic state under such harsh hydrothermal reaction conditions. In situ hydrothermal FTIR spectroscopy was conducted at 250 °C to monitor metal oxidation processes. The interaction of Zn with Co under hydrothermal conditions was examined, and comparisons were made with Fe + Co and Al + Co systems. (Standard electrode potentials of Al, Zn, and Fe are listed in Table S1.) In the Fe + Co system, a Co–O signal emerged after approximately 5 min at 250 °C. For Al + Co, a weak Co–O signal was detected, while no Co–O signal was observed in the Zn + Co system (Fig. S4a). This suggests that Co oxidation is suppressed in the Zn + Co system due to the reductive environment created by Zn under hydrothermal conditions, as evidenced by the significantly higher H_2_ pressure generated with Zn or Al compared to Fe (Fig. S4b). However, slight Co oxidation occurred in the Al + Co system, indicating that a strongly reductive environment alone is insufficient to completely prevent Co oxidation. We propose that hydrogen adsorbed and activated by ZnO further facilitates the reduction of oxidized Co species, ensuring Co remains in its metallic state.

Support for the above assumption was provided by H_2_-TPR analysis of the Co@ZnO catalyst after air exposure (Fig. 4b). For comparison, commercial Co was also analyzed. In the TPR profiles of commercial Co, two well-defined hydrogen uptake peaks were centered at ~ 235 and 300 °C, which corresponded to the well-known Co_3_O_4_ → CoO and CoO → Co transformations, respectively [39, 40]. Notably, these reduction peaks were ~ 100 °C lower in temperature compared to the bulk Co_3_O_4_ reduction profile, probably because only the Co surface was oxidized. In contrast, the two high-temperature reduction peaks of commercial Co were replaced by a single peak in the lower-temperature region in the Co@ZnO catalyst, and this peak was assigned to the overlap of the two-step reduction of Co_3_O_4_ to CoO and Co^°^, a phenomenon also observed when Co supported on Al_2_O_3_ [33, 34]. This indicates the presence of ZnO on the Co surface appeared to facilitate the maintenance of Co in its zero-valence state by enabling hydrogen activation as part of a “refreshment” process. To investigate whether the spatial relationship of Co and ZnO influenced this refreshment process, H_2_-TPR analysis was used to examine a mixture of commercial Co and ZnO. The resulting TPR profile differed from that of Co@ZnO and closely resembled that of commercial Co (Fig. S5). Thus, the supportive structure of Co and ZnO was critical to the refreshment process, as the direct contact between ZnO and Co likely enhanced hydrogen spillover from ZnO to Co.

The influence of the chemical state of Co on CO_2_ methanation was evaluated by comparing the products of CO_2_ reduction using Co@ZnO and commercial Co as catalysts, with gaseous H_2_ as the reductant instead of Zn. As shown in Table S2, the CH_4_ yield with commercial Co was 52% lower than that obtained with Co@ZnO, and undesired by-products such as CO and acetic acid were also generated. In contrast, when Co@ZnO was used as the catalyst, the reaction produced only CH_4_ along with a small amount of formic acid. In Co-based catalytic systems, CoO_x_ species serve as active sites for transforming CO_2_ into CO, and CO, along with formic acid, can combine via carbine insertion to form acetic acid [41]. Thus, the presence of CoO_x_ species on Co-based catalysts often leads to undesirable side reaction pathways that produce CO and acetic acid. However, with the Co@ZnO catalyst, the maintenance of Co in its zero-valent state effectively inhibited the formation of CO and these side reactions. Additionally, the stability of Co during the CO_2_ hydrothermal methanation process was assessed by examining potential Co leaching. Inductively coupled plasma (ICP) analysis revealed that the concentration of Co ions in the solution after the reaction was only 8.57 ppm, indicating that Co leaching was negligible under these conditions.

### Catalytic Properties of Co@ZnO Catalyst in CO_2_ Methanation

**Fig. 5:**
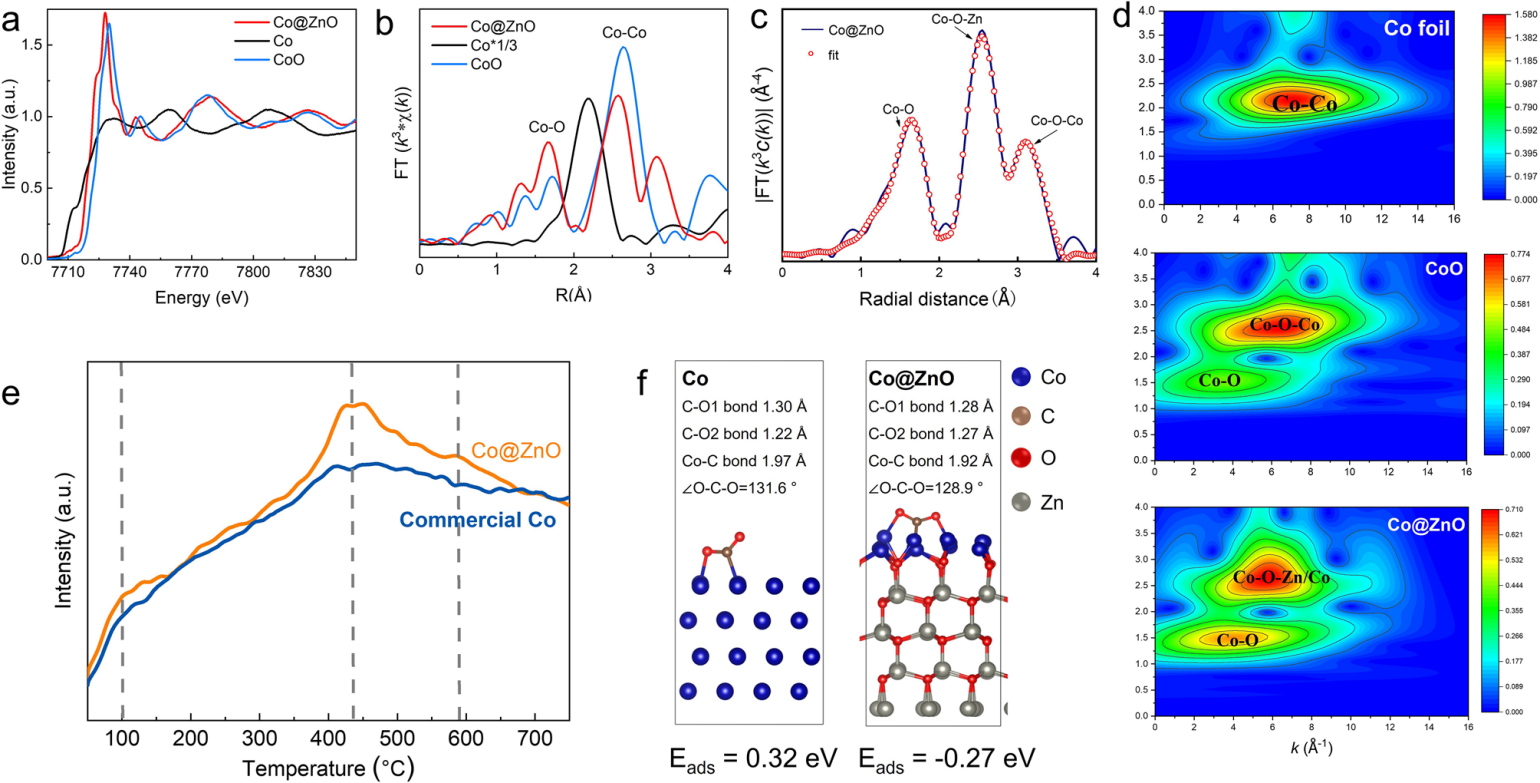
Synchrotron XAFS measurement and catalytic performance of Co@ZnO catalyst. **a** Co K-edge XANES spectra of the Co@ZnO catalyst and reference samples, **b** Fourier transformed (FT) *k*^3^-weighted χ(*k*)-function of the EXAFS spectra for the Co K-edge, **c** Corresponding EXAFS fitting curves at R space, **d** Corresponding WTs for the K^3^-weighted Mn K-edge EXAFS signals of Co@ZnO, Co foil, and CoO, **e** CO_2_-TPD of Co@ZnO catalyst and pre-reduced commercial Co, **f** Comparative DFT calculations of Co@ZnO catalyst and pre-reduced commercial Co

The catalytic performance of in situ formed Co@ZnO catalyst was investigated by exploring the nature of the metal–metal oxide interfaces of Co@ZnO catalyst and how ZnO tunes the properties of Co sites. Element-selective X-ray absorption fine structure (XAFS) measurements at the Co K-edge were performed to determine the chemical state and coordination environment of Co species at the atomic level (Fig. 5). The normalized X-ray absorption near edge structure (XANES) curves of the Co K-edge for Co@ZnO, Co foil, and CoO showed that the absorption edge of Co@ZnO was located between those of CoO and Co, suggesting that Co in Co@ZnO had a positive valence state (0 < δ < 2; Fig. 5a). This deviation from a zero-valent state is likely due to the growth of ZnO on the Co surface, which alters the electronic structure of Co at the Co − ZnO interface. Indeed, a similar pre-edge peak around 7723 eV in the XANES curves of Co-doped ZnO has been reported in previous studies [42]. Additionally, the main peaks in the Co@ZnO spectrum were found to be shorter than the Co–O and Co–Co peaks observed in CoO (Fig. 5b). Further analytical scrutiny through wavelet transforms (WT) analysis of EXAFS for Co foil, CoO, and Co@ZnO was executed (Fig. 5d), with the Co@ZnO revealing the high signal intensity in the middle of the plot, especially in the red region, likely represents Co–O coordination. Additionally, another signal peak related to other elements’ coordination was observed, suggesting that Zn is present in sample [43]. These signals show a more complex local structural environment than pure Co foil. The coordination configuration of the Co atom in Co@ZnO was further analyzed through quantitative EXAFS curve fitting (Fig. 5c). The best-fit analysis revealed a primary peak corresponding to the Co–O–Zn/Co configuration (Fig. S6, Table S3). Based on the experimental XAFS spectra and the EXAFS fitting data, we propose that a Co–O–Zn/Co local atomic structure is formed at the Co@ZnO interface, which is likely to influence the catalytic activity of the Co core, as discussed in the subsequent sections.

CO_2_-TPD analysis was performed to assess the CO_2_ chemisorption capabilities of the Co@ZnO catalyst, which is the key initial step in CO_2_ methanation. For comparison, commercial pre-reduced Co was also tested. As shown in Fig. 5e, both samples exhibited three peaks at approximately 100, 400, and 600 °C, corresponding to the desorption of CO_2_ from weak, medium, and strong adsorption sites, respectively. However, the peak intensities for the Co@ZnO catalyst were significantly higher than those for the commercial Co, indicating a superior capacity for CO_2_ adsorption in the Co@ZnO catalyst. Moreover, the CO_2_-TPD profile of Co@ZnO displayed a broad desorption band between 370 and 600 °C, suggesting enhanced chemisorption of CO_2_ on moderately basic sites. Since medium-strength CO_2_ chemisorption is known to promote higher reaction activity in CO_2_ methanation [44–46], the abundance of moderately basic sites on Co@ZnO likely contributes to its increased catalytic activity in CO_2_ methanation.

To better understand the mechanism behind the enhanced CO_2_ adsorption ability of the Co@ZnO catalyst, comparative density functional theory (DFT) calculations were conducted on CO_2_ adsorption over Co@ZnO and Co nanoparticles. Figure 5f illustrates the CO_2_ adsorption structures on both catalysts. For Co nanoparticles, the CO_2_ binding distance was estimated to be approximately 1.97 Å, with an adsorption energy of 0.32 eV. In contrast, for Co@ZnO, the C–Co bond length was found to be around 1.92 Å, and notably, the CO_2_ adsorption energy on Co@ZnO was much lower, at − 0.27 eV. This much lower value was ascribed to the electron redistribution effect of ZnO on Co surface (Fig. S7). When CO_2_ adsorbs on the surface of Co@ZnO, the electron density is more evenly distributed from ZnO to Co, resulting in a more stable adsorption configuration. Thus, with the growth of ZnO on the Co, Co would exhibit the enhanced chemisorption of CO_2_, leading to deeper CO_2_ reduction for efficient CH_4_ formation.

### Mechanistic Study of CO_2_ Methanation by In situ Hydrothermal FTIR

We further investigated the mechanism of CO_2_ methanation in the hydrothermal system by analyzing the reaction products. As shown in Fig. S8a, the gaseous products were predominantly CH_4_, with only small amounts of unreacted CO_2_ and H_2_ detected. Notably, CO was not observed in the product stream. Furthermore, FID analysis of the gaseous products revealed negligible amounts of C_2_H_6_ and C_3_H_8_ (Fig. S8b), with their production being effectively suppressed as the amount of Zn was increased. Gas chromatography–mass spectrometry (GC–MS) analysis of the liquid products revealed only trace amounts of formic acid (Fig. S8c). This narrow product distribution suggests that CO_2_ hydrothermal methanation with the Co@ZnO catalyst is highly selective, surpassing previous reports on Co-based catalysts for CO_2_ methanation, where CO and multi-carbon by-products were typically generated.

**Fig. 6:**
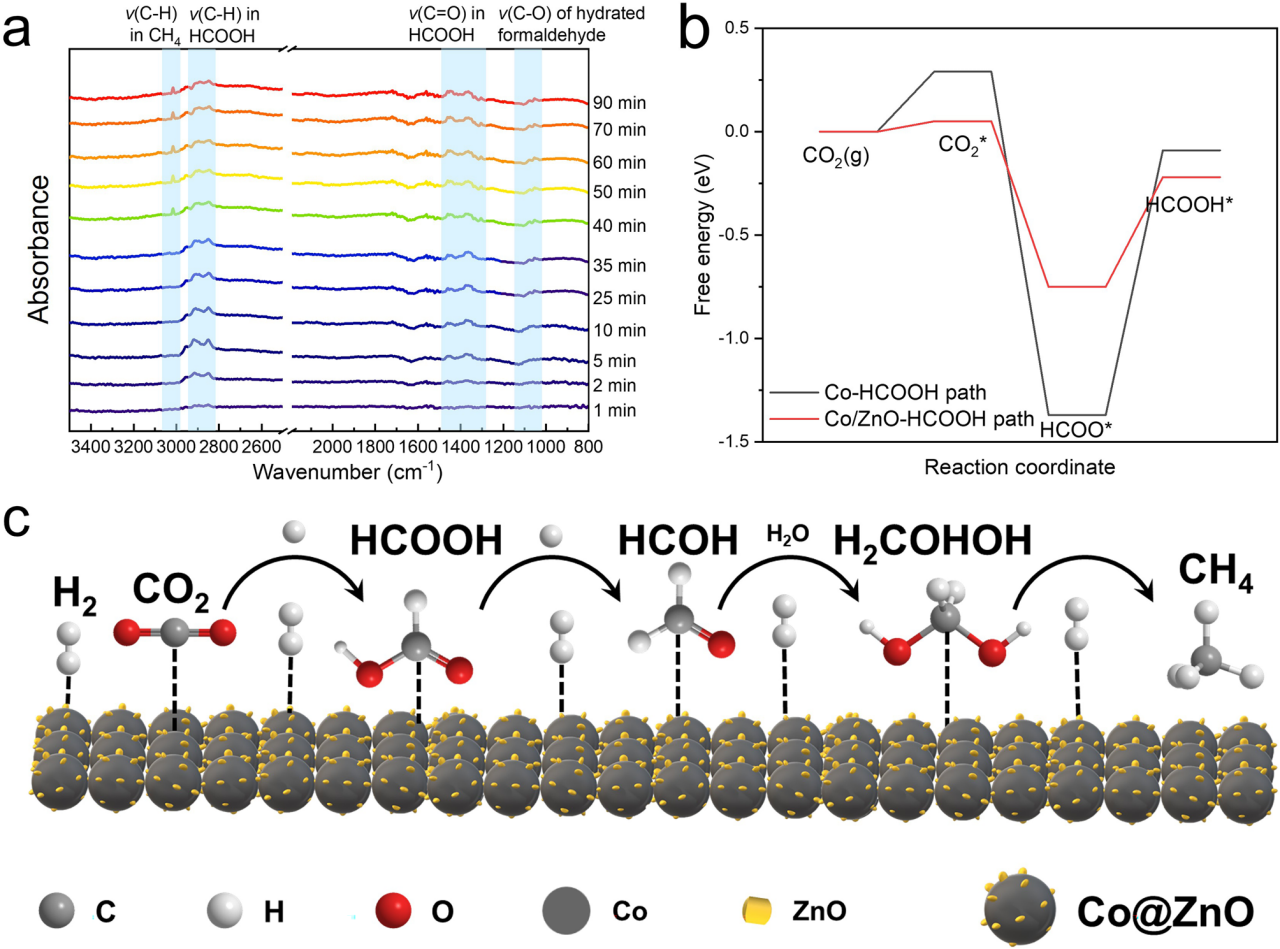
In situ FTIR study and proposed mechanism. **a** In situ hydrothermal FTIR spectra of CO_2_ methanation with Zn and Co, **b** reaction energy profile of HCOOH as the intermediate, **c** schematic illustration of the proposed reaction mechanism

Further insight into CO_2_ hydrothermal methanation was gained by monitoring the reaction in real-time using in situ hydrothermal FTIR (Fig. 6a). For comparison, CO_2_ hydrogenation using only Zn as the reductant was also tracked. The CO_2_ methanation with Zn and Co was evaluated at 250 °C for 90 min, revealing that formic acid was efficiently produced as the first product starting around 2 min, with its concentration steadily increasing thereafter (Fig. 6a). However, at approximately 25 min, the typical C − H stretching vibrations (*v*(C − H)) of formic acid at 2920 and 2840 cm^−1^ showed multiple peaks, indicating the formation of formaldehyde (HCOH) [47]. Additionally, two smaller peaks emerged around 1050 cm^−1^. By comparing with FTIR spectra of HCOH under hydrothermal conditions (Fig. S9), these signals were attributed to the C − O stretch of hydrated formaldehyde. As the reaction progressed, CH_4_ was produced, with a notable increase in the peak intensity at 3014 cm^−1^ starting at 45 min, which aligned with the sharp rise in CH_4_ formation observed after 30 min in the temporal analysis of CO_2_ hydrothermal methanation products (Fig. S3). The absence of the characteristic CO peak at 2170 cm^−1^ confirmed that CO was not formed during the reaction. Furthermore, formic acid acted as an intermediate, exhibiting a dynamic equilibrium between its generation and subsequent transformation. These results suggest that formic acid was the initial intermediate, which then converted to HCOH, ultimately leading to CH_4_ production. To further validate this conclusion, formic acid and CO were individually tested for CH_4_ production under the same reaction conditions. As anticipated, a higher yield of CH_4_ was obtained from formic acid compared to CO, as detailed in Table S4.

In contrast, when only Zn was used for CO_2_ hydrothermal reduction, formic acid was also produced at around 2 min (Fig. S9b). However, despite the extended reaction time, no CH_4_ was formed, and only formic acid accumulated, highlighting the necessity of Co for efficient methane production. In addition, even with ZnO simultaneously applied as the catalyst, only formic acid was formed with prolonged reaction time (Fig. S9c).

To gain a deeper insight in the reaction mechanism, density functional theory (DFT) calculations were performed. Given the formation of a Co–O–Zn/Co local atomic structure on the Co@ZnO catalyst surface, which indicates a strong metal-support interaction (SMSI) effect between Co and ZnO, additional calculations were conducted for a system using Co alone as the catalyst for comparison. The calculations simulated the reaction pathway for CO₂ reduction to HCOOH, with the pathway involving CO as an intermediate used as a reference for comparison (Fig. S10). As illustrated in Figs. 6b and S10b, the energy barrier for the rate-determining step of HCOOH formation was calculated to be 0.53 eV, whereas for CO formation, it was 0.68 eV. This indicates that the HCOOH formation pathway is thermodynamically more favorable compared to the pathway involving CO as an intermediate. Moreover, due to its Co–O–Zn/Co local atomic structure, the Co@ZnO catalyst exhibited superior catalytic activity for CO₂ reduction compared to Co alone, confirming the presence of the SMSI effect between Co and ZnO.

Based on the in situ hydrothermal FTIR results, a proposed mechanism for CO_2_ hydrothermal reduction to CH_4_ with Zn and Co is outlined in Fig. 6c. Under hydrothermal conditions, Zn is oxidized to generate H_2_, which then reduces CO_2_ to formic acid while forming ZnO. ZnO acts as a hydrogen reservoir, dissociating H_2_ for further reduction of formic acid. The hydrogen atoms diffuse efficiently to the Co surface, facilitating the sequential formation of HCOH and CH_4_. Ultimately, CH_4_ is selectively produced and desorbed from the catalyst surface, with H_2_O generated as a by-product.

Based on the proposed mechanism, the formation of formic acid is a key step in selective CO_2_ methanation. Since the conversion of CO_2_ to formic acid relies on water splitting for hydrogen production from Zn, we sought to enhance CH_4_ yield by increasing the amount of Zn or raising the reaction temperature, as higher temperatures promote more efficient water splitting. The effect of Co quantity on CH_4_ production was also examined. Optimal CH_4_ yield (100%) was achieved when CO_2_ methanation was conducted at 300 °C for 2 h with 90 mmol Zn and 40 mmol Co (Fig. S11). Additionally, the stability of the in situ formed Co@ZnO catalyst was demonstrated by repeating the CO_2_ hydrothermal methanation five times or prolonging the reaction time to 10 h. The post-reaction catalysts were then applied to TEM and XRD analysis, which showed almost the same structure as the newly formed Co@ZnO catalyst (Fig. S12). Furthermore, various transition metal catalysts (Fe, Ni, Cu) and noble metal-based catalysts (Pd/C, Pt/C) have been explored for CO_2_ methanation under hydrothermal conditions. However, CH_4_ yields remained below 30% (Table S5), even when using Ni, the most common catalyst for this reaction. These findings highlight the exceptional efficiency of the in situ formed Co@ZnO catalyst.

One of the primary challenges in applying hydrothermal technology is the requirement for high reaction temperatures and pressures. To evaluate the feasibility of Zn-enabled CO_2_ methanation, we analyzed the energy flow of the reaction. In this hydrothermal system, 535.628 kJ of energy can be harnessed per mole of reaction. By accounting for the energy required to heat the reactants, we determined that after three moles of reaction, the energy input for heating is fully compensated. Beyond this point, additional energy can be collected. (Details of the energy flow calculations are provided in the SI.) These energy balance calculations suggest that this CO_2_ methanation process is energetically favorable.

## Conclusions

Efficient CO_2_ methanation was achieved using the in situ generated Co@ZnO catalyst in a one-pot reaction. The reductive environment provided by Zn oxidation and the growth of ZnO on the Co surface helped maintain Co in a stable zero-valence state, overcoming the common issue of Co-based catalyst deactivation due to oxidation. This approach also suppressed the formation of CO and other undesirable by-products, resulting in a 100% CH_4_ yield under optimized conditions. The enhanced catalytic activity of Co@ZnO is attributed to the ZnO-induced modulation of the electronic environment around Co, which facilitates highly efficient CO_2_ activation. By using Zn as a reductant to indirectly harness solar energy and employing a stable Co-based catalyst, this simple, green hydrothermal method for CO_2_ reduction avoids the need for gaseous H_2_ supply or complex catalyst design, offering significant potential for practical applications.

## Supplementary Information

Below is the link to the electronic supplementary material.

Supplementary file1 (DOCX 5655 kb)
