# Supplementary material for: 100% Conversion of CO2–CH4 with Non-Precious Co@ZnO Catalyst in Hot Water
Source: Nanomicro Lett. 2025 Apr 14;17:216. doi: 10.1007/s40820-025-01711-6 (PMC11996728; doi:10.1007/s40820-025-01711-6)
Supplement: Supplementary file 1 — Supplementary file1 (DOCX 5655 kb) [file 40820_2025_1711_MOESM1_ESM.docx]

Supporting Information for

**100% Conversion of CO_2_ to CH_4_ with Non-Precious Co@ZnO Catalyst in Hot Water**

Yang Yang^1#^, Xu Liu^1#^, Daoping He^2^*, and Fangming Jin^1,3,4^*

^1^School of Environmental Science and Engineering, Shanghai Jiao Tong University, Shanghai 200240, P. R. China

^2^China-UK Low-carbon College, Shanghai Jiao Tong University, Shanghai 200240, P. R. China

^3^Shanghai Key Laboratory of Hydrogen Science & Center of Hydrogen Science, State Key Laboratory of Metal Matrix Composites, Shanghai Jiao Tong University, Shanghai 200240, P. R. China

^4^School of Environmental Science and Engineering, Hainan University, Haikou 570228, P. R. China

^#^ Yang Yang and Xu Liu contributed equally to this work.

*Corresponding authors. E-mail: [hedaoping@sjtu.edu.cn](mailto:hedaoping@sjtu.edu.cn) (Daoping He), [fmjin@sjtu.edu.cn](mailto:fmjin@sjtu.edu.cn) (Fangming Jin)

**Supplementary Test S1**

***Distribution of carbon species at 300 °C***

Initially 1.5 MPa CO_2_ was sealed in the reactor.

Henry’s law states:

$$C_{{CO}_{2}=\frac{P_{{CO}_{2}}}{K_{H}}}$$

The Henry's law constant k_H_ changes significantly with temperature, and the solubility of gases decreases at higher temperatures.

At 25 °C, k_H_ ≈ 0.033 mol/(L•atm).

At 300 °C, water approaches a supercritical state, and the solubility of gases becomes very low. k_H_ may increase by several orders of magnitude, making the dissolved CO₂ concentration much smaller than at room temperature.

Assuming k_H_ at 300 °C is 10 times the value at room temperature (i.e. k_H_≈0.33 mol/(L•atm)). Thus, with 1.5 MPa CO_2_ sealed at room temperature, the concentration of CO_2_ in the solution at 300 °C can be calculated as:

$$C_{{CO}_{2}=\frac{P_{{CO}_{2}}}{K_{H}}=\frac{1.5*9.87}{0.33}=0.045 mol/L}$$

With CO_2_ dissolved in water, the following reactions occur:

CO_2_+H_2_O⇌H_2_CO_3_

H_2_CO_3_⇌H^+^+HCO_3_^−^(K_a1_)

HCO_3_^−^⇌H^+^+CO_3_^2−^(K_a2_)

At high temperatures, both K_a1_ and K_a2_ increase, but the alkaline environment (due to NaOH) greatly suppresses the concentration of H_2_CO_3_.

Thus, when no NaOH was added to the solution, the distribution of carbon species is: [H_2_CO_3_] ≈ $0.045 mol/L$; [HCO_3_^−^] ≈ 0.

When 0.1 mol/L NaOH was added, the distribution of carbon species is: [H_2_CO_3_] ≈ $0$; [HCO_3_^−^] ≈ $0.045 mol/L$.

When 0.2 mol/L NaOH was added, the distribution of carbon species is: [H_2_CO_3_] ≈ $0$; [HCO_3_^−^] ≈ $0 mol/L$; [CO_3_^2−^] ≈ $0.045 mol/L$.

When 0.3 mol/L NaOH was added, the distribution of carbon species is: [H_2_CO_3_] ≈ $0$; [HCO_3_^−^] ≈ $0 mol/L$; [CO_3_^2−^] ≈ $0.045 mol/L$.

**Supplementary Test S2**

***Energy requirements accounting***

The reaction is divided into 3 parts to consider. 1) Energy Input for Heating: The first part involves calculating the energy required to heat all the substances to the desired temperature. 2) Enthalpy Changes of the Reaction: The second part focuses on evaluating the enthalpy changes during the reaction, which reflect the heat absorbed or released by the system. 3) Energy Recovery: The third part accounts for the energy recovered from the reaction.

***Step 1: Energy Inpu***t

Take 1 mol reaction as an example. According to the typical reaction settings, we need to calculate the heat required to heat a mixture containing 1082.5 mL of water, 2577.3 mmol of cobalt (Co), 3866 mmol of zinc (Zn), and 1 mol of carbon dioxide (CO₂) from 20°C to 300°C (the amount of water is the entire water added to the reaction system). The following constant numbers are used.

Specific heat capacity of water: c_water_≈4.18 J/g

Specific heat capacity of cobalt: c_Co_≈0.421 J/g

Specific heat capacity of zinc: c_Zn_≈0.388 J/g

Heat absorbed by each substance:

Q_water_=m_water_⋅c_water_⋅ΔT

Q_Co_=m_Co_⋅c_Co_⋅ΔT

Q_Zn_=m_Zn_⋅c_Zn_⋅ΔT

For a closed system, the heat capacity of CO₂ should be considered:

Molar heat capacity of CO₂ at constant volume Cv≈28.46 J/mol

Heat absorbed by CO₂:

Q_CO2_=n_CO2_×Cv×ΔT

Q_total_=Q_water_+Q_Co_+Q_Zn_+Q_CO2_

Thus, the total heat required to heat this closed system from 20°C to 300°C is approximately 1320.2 kJ.

***Step 2: Enthalpy change***

We calculated the enthalpy change of the reaction as follow:

4Zn + 2H_2_O + CO_2_(g) = CH_4_(g) + 4ZnO

| T (^o^C) | Delta H (kJ) | Delta S (J/k) | Delta G (kJ) |
| --- | --- | --- | --- |
| 100 | -517.909 | -180.592 | -450.521 |
| 200 | -526.020 | -199.845 | -431.464 |
| 300 | -535.628 | -218.166 | -410.586 |
| 400 | -550.269 | -241.656 | -387.599 |

At 300 ^o^C, the typical reaction temperature, the enthalpy change of the reaction is -535.628 kJ, indicating that it is an exothermic reaction.

***Step 3: Energy recovered from the reaction***

Since the reaction is exothermic, we tried to investigate whether the energy needed for heating up the reaction system can be compensated from the reaction itself in case the reaction system is kept adiabatic.

For every new mole of reaction, we need to calculate the heat required to heat a mixture containing 36 mL of water, 2577.3 mmol of cobalt (Co), 3866 mmol of zinc (Zn), and 1 mol of carbon dioxide (CO₂) from 20°C to 300°C. The constant numbers are the same to that in Step 1.

Thus, the total heat required to heat substances for 1 mole of reaction from 20°C to 300°C is approximately 98.1 kJ. Consequently, for every mole of reaction, 535.628-98.1=437.5 kJ energy can be collected.

Since in the initial step, for 1 mole of reaction, 1320.2 kJ energy input is needed, thus, after 1320.2/437.5=3 mole of reaction, the energy for heating the reaction system can be compensated, and after that, more energy can be collected from the reaction system.

Based on these results, if an adiabatic reaction system is applied, after 3 moles of reaction, no more energy is needed to keep the reaction running, while it can sustain itself sufficiently.

**Supplementary Figures**

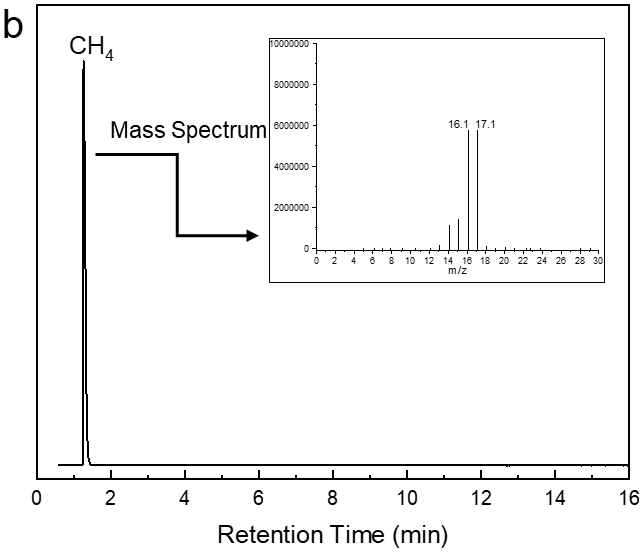


**Fig. S1** (**a**) Effect of NaOH concentration on CH_4_ yield and (**b**) GC-MS analysis of gaseous products after isotope reaction with ^13^CO_2_ (reaction conditions: 1.5 MPa CO_2_ or 1 MPa ^13^CO_2_, 60 mmol Zn, 40 mmol Co, 2 h, 300 ℃)


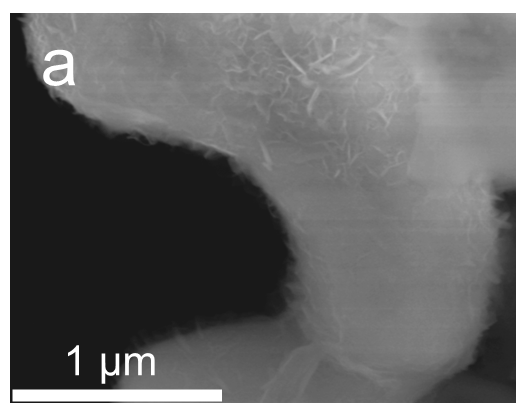

**Fig. S2** SEM image (**a**) and XRD patterns (**b**) of Co@ZnO catalyst after washing away ZnO

**Fig. S3** The variation of CH_4_ yield with reaction time (reaction conditions: 60 mmol Zn, 40 mmol Co, 1.5 MPa CO_2_, 0.1 mol/L NaOH, 300 °C)

**Fig. S4** In-situ hydrothermal FTIR (**a**) and hydrogen pressure (**b**) of Zn+Co, Fe+Co, and Al+Co reaction under hydrothermal conditions

**Fig. S5** H_2_-TPR profiles of Co@ZnO catalyst, commercial Co, and commercial Co mixed with ZnO


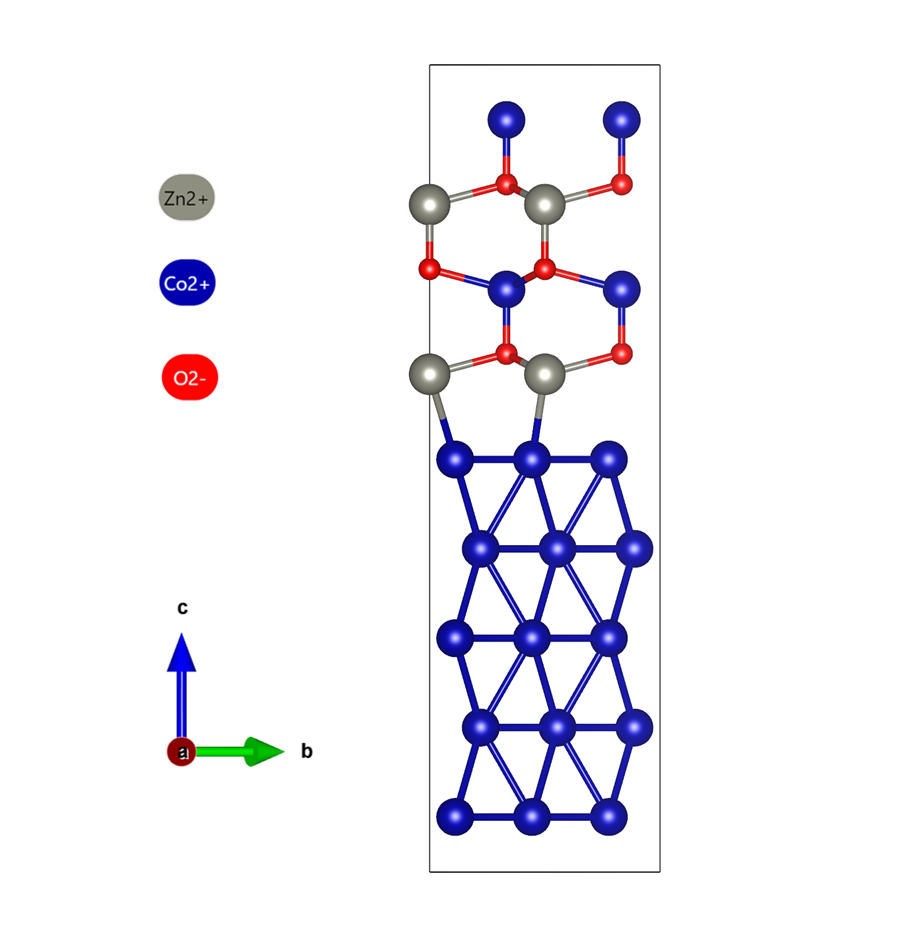


**Fig. S6** Schematic model of Co@ZnO catalyst


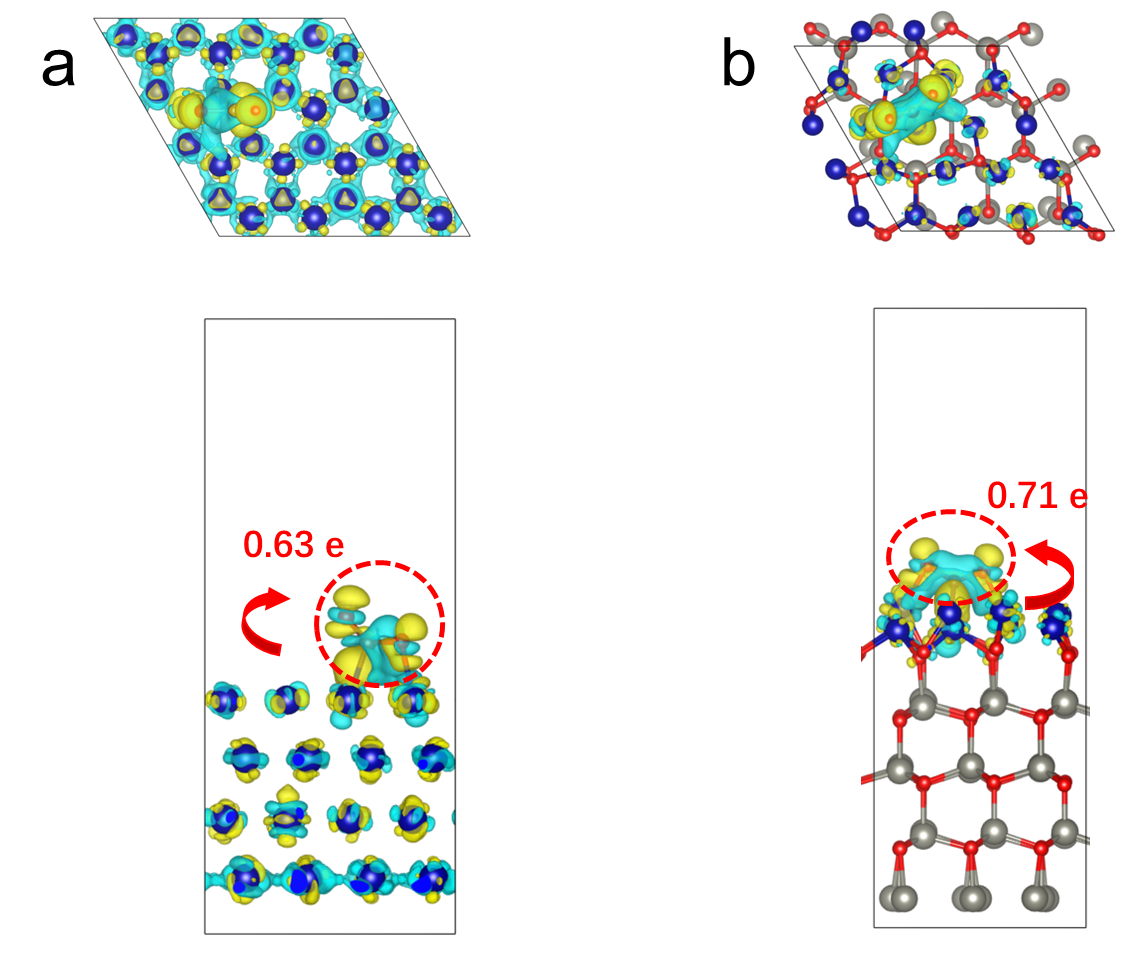


**Fig. S7** Charge density difference and bader charge transfer between CO_2_ and Co (**a**) and Co@ZnO (**b**) (blue, yellow, red, and gray balls represent Co, C, O, and Zn atoms, respectively; yellow and blue clouds represent increased or decreased charge, respectively)

**Fig. S8** TCD (**a**) and FID (**b**) analysis of gaseous samples and GC-MS analysis of liquid samples (**c**) from CO_2_ hydrothermal methanation (reaction conditions: 60 mmol Zn, 40 mmol Co, 1.5 MPa CO_2_, 0.1 mol/L NaOH)

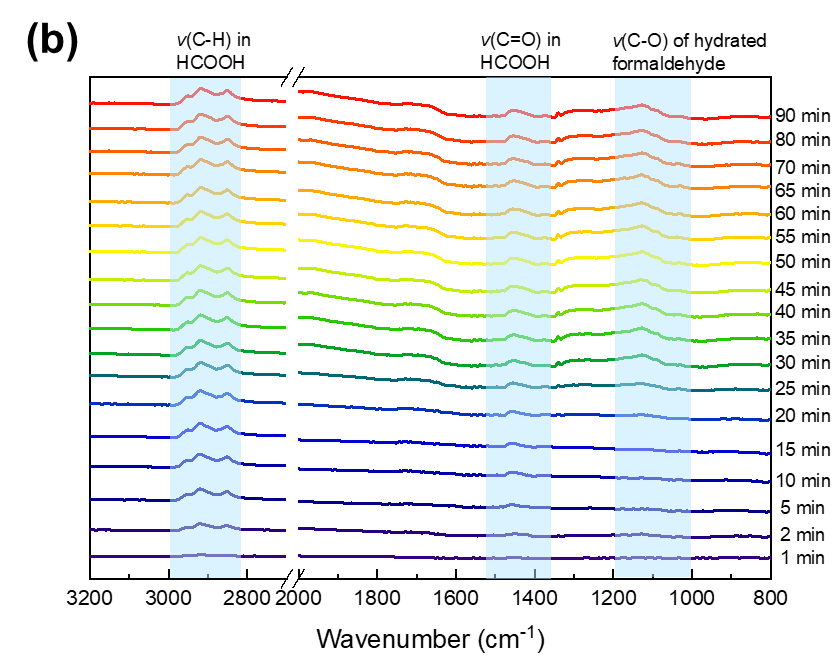

**Fig. S9** In-situ hydrothermal FTIR of formaldehyde (**a**), CO_2_ methanation with Zn as the reductant (**b**), and CO_2_ methanation with Zn as the reductant via ZnO as the catalyst (**c**)


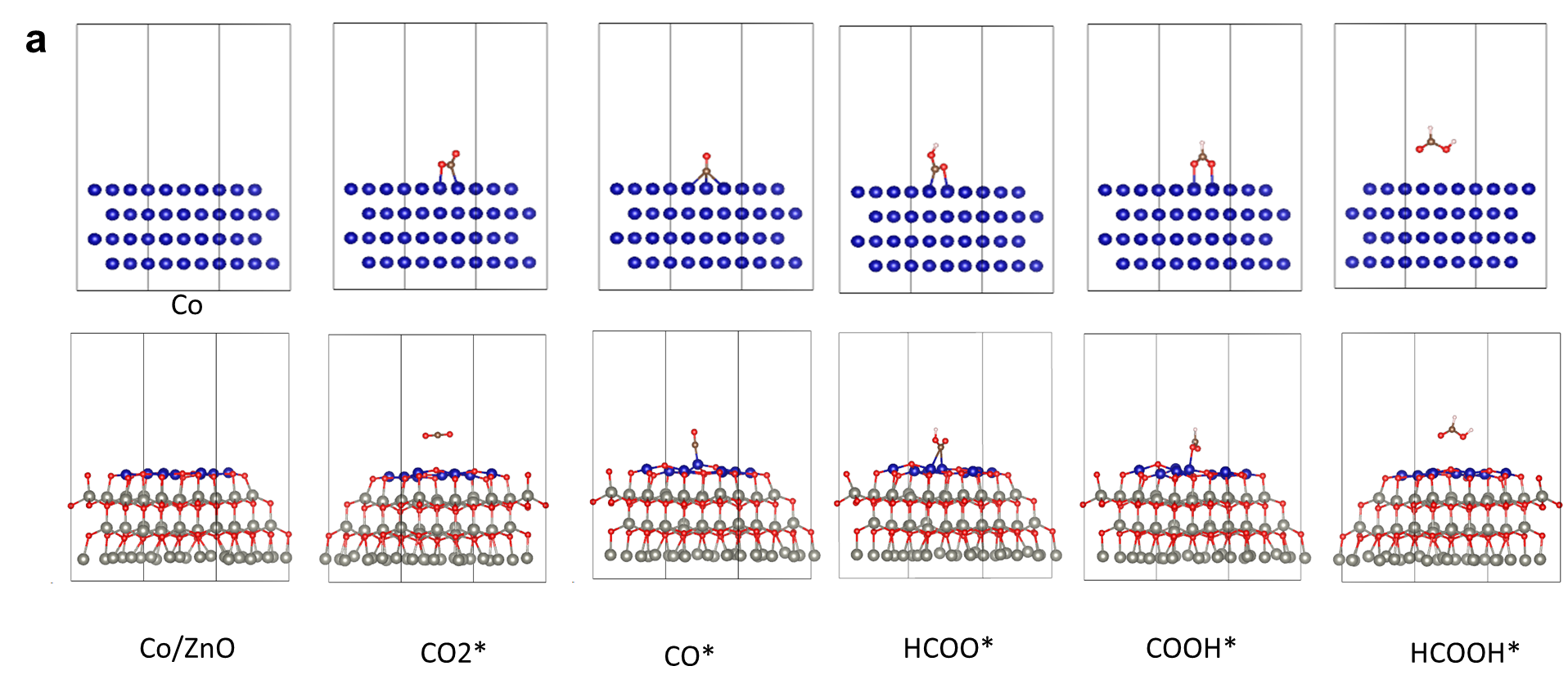

**Fig. S10** Geometrically optimized structure of Co or Co@ZnO (**a**) and reaction energy profile of CO as the intermediate (**b**)

**Fig. S11** Optimization of CO_2_ hydrothermal methanation by altering different parameters. (**a**) Zn quantity. (**b**) Reaction temperature. (**c**) Co quantity (reaction conditions: 1.5 MPa CO_2_, (**a**) 300 °C, 2 h, 40 mmol Co, 0.1 mol/L NaOH, (**b**) 60 mmol Zn, 2 h, 40 mmol Co, 0.1 mol/L NaOH, (**c**) 300 °C, 2 h, 60 mmol Zn, 0.1 mol/L NaOH)


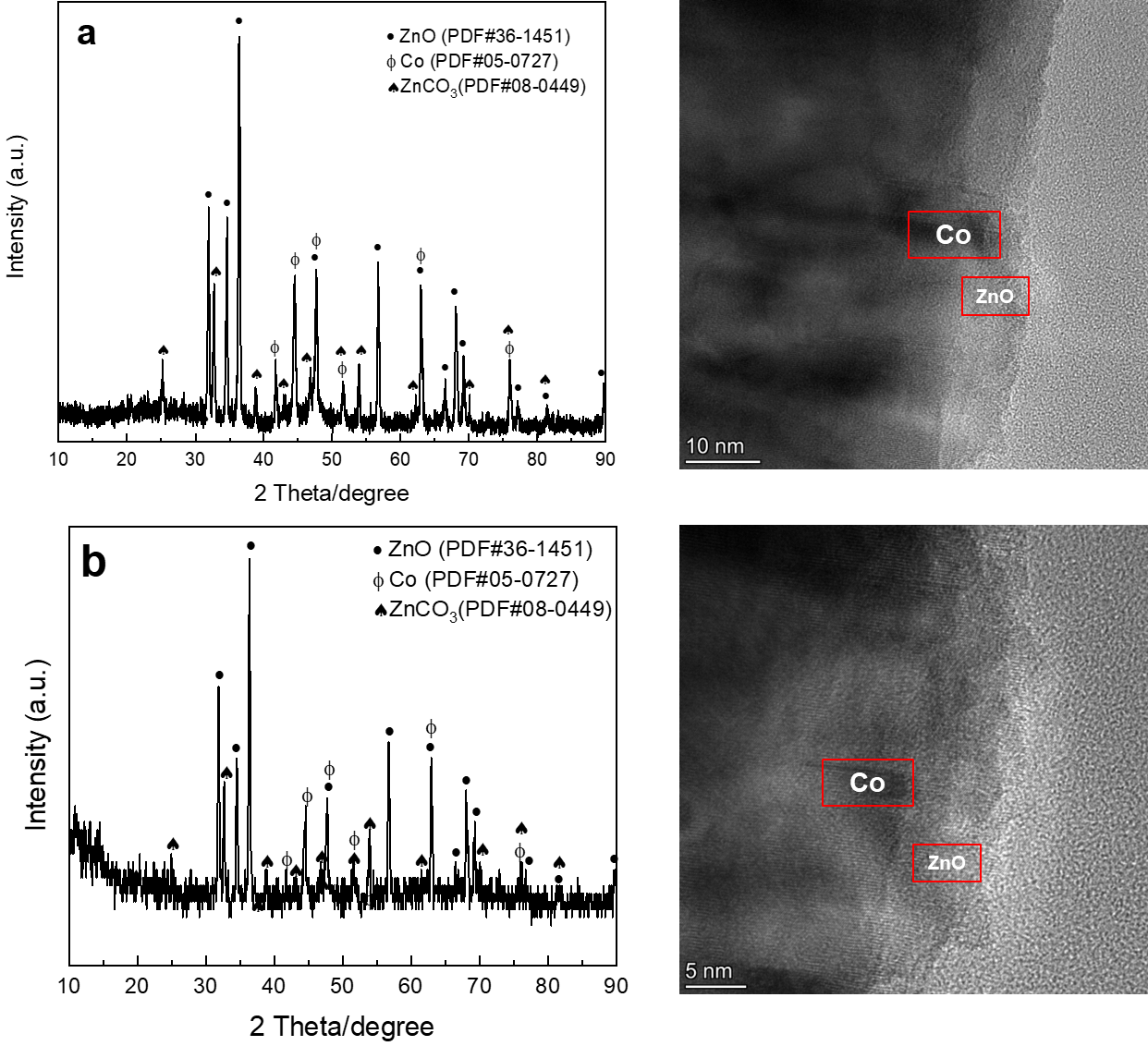


**Fig. S12** XRD and TEM analysis of catalysts after 5 times reuse (**a**) or 10 h reaction (**b**)


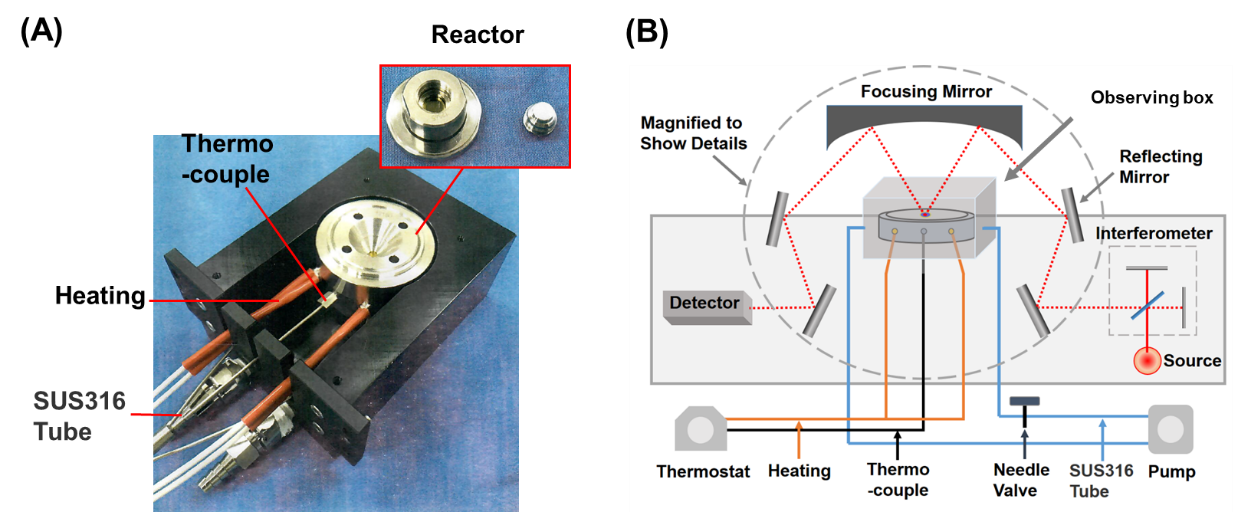


**Fig. S13** (**A**) Image of the high-pressure/high-temperature reactor. (**B**) Schematic drawing of the in-situ hydrothermal FTIR system

**Supplementary Tables**

| **Table S1** Standard electrode potentials of Al, Zn, and Fe | |
| --- | --- |
| **Metal** | **φA/V** |
| Al | -1.662 |
| Zn | -0.762 |
| Fe | -0.447 |

| **Table S2** Product distribution of CO_2_ hydrothermal reduction with Co@ZnO or commercial Co as the catalyst and the corresponding product yields^a^ | | | | |
| --- | --- | --- | --- | --- |
| **Catalyst** | **Product distribution and yield** | | | |
|  | CH_4_ | CO | Formic acid | Acetic acid |
| Co@ZnO | 44.7% | - | 2.3% | - |
| Commercial Co | 21.3% | 7.8% | 3.6% | 4.5% |

^a^Reaction conditions: 1.5 MPa CO_2_, 3.5 MPa H_2_, 300 °C, 2 h, 0.1 mol/L NaOH.

| **Table S3** EXAFS data fitting results of Co@ZnO * | | | | | | |
| --- | --- | --- | --- | --- | --- | --- |
| Sample | Path | *CN^a^* | *R*(Å)*^b^* | *σ*^2^ (Å^2^)*^c^* | Δ*E*_0_(eV)*^d^* | *R* factor |
| Co K-edge (*Ѕ*_0_^2^=0.764) | | | | | | |
| Co foil | Co-Co | 12.0* | 2.492±0.001 | 0.0063 | 7.7±0.3 | 0.0013 |
| CoO | Co-O | 6.4±0.7 | 2.114±0.011 | 0.0108 | -1.1±0.9 | 0.0111 |
|  | Co-O-Co | 14.3±0.5 | 3.012±0.006 | 0.0093 |  |  |
| sample_Co | Co-O | 6.2±0.3 | 2.068±0.008 | 0.0065 | 0.5±1.4 | 0.0048 |
|  | Co-O-Zn | 6.5±0.2 | 2.934±0.005 | 0.0106 | -5.0±0.7 |  |
|  | Co-O-Co | 7.5±0.4 | 3.471±0.007 |  |  |  |

*^a^CN*, coordination number; *^b^R*, the distance between absorber and backscatter atoms; *^c^σ*^2^, the Debye Waller factor value; *^d^ΔE*_0_, inner potential correction to account for the difference in the inner potential between the sample and the reference compound; *R* factor indicates the goodness of the fit. *S*0^2^ was fixed to 0.764, according to the experimental EXAFS fit of Co foil by fixing *CN* as the known crystallographic value. * This value was fixed during EXAFS fitting, based on the known structure of Co. Fitting conditions: *k* range：3.0 - 12.0; *R* range: 1.0-3.5; fitting space: R space; *k*-weight = 3. A reasonable range of EXAFS fitting parameters: 0.700 < *Ѕ*_0_^2^ < 1.000; *CN >* 0; *σ*^2^ > 0 Å^2^; |Δ*E*_0_| < 15 eV; *R* factor < 0.02.

| **Table S4** Product distribution of HCOOH or CO hydrothermal reduction with Zn and Co^a^ | | | | | | |
| --- | --- | --- | --- | --- | --- | --- |
| **Substrate** | **Product distribution and yield** | | | | | |
|  | CH_4_ | C_2_H_6_ | C_3_H_8_ | C_4_H_10_ | C_5_H_12_ | C_6_H_14_ |
| HCOOH | 71.8% | 4.2% | 3.9% | 2.5% | 1.7% | 1.1% |
| CO | 60.0% | 1.7% | 0.9% | 0.5% | 0.3% | 0.2% |

^a^Reaction conditions: 0.9 mol/L HCOOH (the carbon content was identical to 1.5 MPa CO) or 1.5 MPa CO, 60 mmol Zn, 40 mmol Co, 300 °C, 2 h, 0.1 mol/L NaOH.

| **Table S5** The yield of CH_4_ with different catalysts^a^ | | | |
| --- | --- | --- | --- |
| Entry | Reductant | Catalyst | The yield of CH_4_ (%) |
| 1 | Zn | Co | 99.9 |
| 2 | Zn | Fe | 2.19 |
| 3 | Zn | Cu | 9.76 |
| 4 | Zn | Ni | 24.12 |
| 5 | Zn | Pd/C | 29.44 |
| 6 | Zn | Pt/C | 4.23 |

^a^Reaction conditions: 1.5 MPa CO_2_, 90 mmol Zn, 40 mmol catalyst, 300 °C, 2 h, 0.1 mol/L NaOH.
